# Platelet-leukocyte aggregate is associated with adverse events after surgical intervention for rheumatic heart disease

**DOI:** 10.1038/s41598-019-49253-3

**Published:** 2019-09-10

**Authors:** Chaonan Liu, Yang Yang, Lei Du, Si Chen, Jie Zhang, Chongwei Zhang, Jing Zhou

**Affiliations:** 1 Department of Laboratory Medicine, West China Hospital, Sichuan University, Chengdu, China; 2 Department of Anesthesiology, West China Hospital, Sichuan University, Chengdu, China; 3 Key Laboratory of Transplant Engineering and Immunology, West China Hospital, Sichuan University, Chengdu, China; 4 Present Address: Department of Anesthesiology, Affiliated Hospital of Guiyang Medical College, Guiyang, China

**Keywords:** Peripheral vascular disease, Risk factors

## Abstract

Platelet-leukocyte aggregate (PLA) is implicated in the etiology of both vascular lesions and cardiovascular events. This prospective cohort study aimed to examine the prognostic value of PLA for major adverse cardiac and cerebrovascular events (MACCE) and perioperative adverse events (AEs) in patients with rheumatic heart disease undergoing surgical intervention by Cox proportional hazard regression and logistic regression. A total of 244 patients were included, of whom 7 were lost to follow-up. Among the analyzed 237 subjects who completed 3-year follow-up, 30 experienced MACCE and 38 experienced perioperative AEs. Preoperative PLA was higher in subjects who developed MACCE (13.32%) than in those who did not (8.69%, p = 0.040). In multivariate regression, elevated PLA was associated with increased MACCE (hazard ratio 1.51 for each quartile, 95% CI 1.07–2.13; p = 0.020), and perioperative AEs (odds ratio 1.61, 95% CI 1.14–2.26; p = 0.007). The optimal PLA cut-off for predicting MACCE was 6.8%. Subjects with PLA > 6.8% had a higher prevalence of MACCE (17.1% vs. 5.5%, p = 0.009) and perioperative AEs (19.9% vs. 8.6%, p = 0.018). Kaplan-Meier analysis showed shorter MACCE-free survival in patients with PLA > 6.8% (p = 0.007, log rank). Elevated preoperative PLA is associated with increased MACCE and perioperative AEs in patients with rheumatic valve disease undergoing surgical intervention.

## Introduction

Cardio-cerebrovascular disorders resulting from acute or chronic inflammation of the vascular wall may lead to severe cardiac and cerebrovascular events^1–4^. Although the mechanism of these disorders remains poorly understood, activation of innate immune cells (e.g., monocytes and neutrophils) and adaptive immune cells (e.g., lymphocytes and plasma cells) is believed to be involved^5–8^, through regulation by a complex network of cytokines and chemokines such as tumor necrosis factor (TNF)-α^9^. Platelets can bind to activated leukocytes via the selectin ligand PSGL-1 to form platelet-leukocyte aggregates (PLA)^10^, which drive the migration of leukocytes to endothelium, where they can cause vascular lesions^7,11^. Clinical studies have shown a higher PLA level in patients with unstable angina and acute coronary syndrome than in those with stable angina^12,13^. Moreover, circulating PLA is higher in patients with acute myocardial infarction or ischemic stroke than in control subjects^10,12–17^. In animal studies, blocking PLA formation can inhibit the initiation, development and progression of atherosclerosis^18^, as well as reduce myocardial and cerebral ischemia/reperfusion injury^15^. These studies suggest a correlation between vascular lesions and PLA, but clinical evidence on such a correlation is lacking.

We hypothesized that PLA is associated with increased major cardiac and cerebrovascular events (MACCE) following cardiac surgery. We tested this possibility in a cohort of patients undergoing surgical intervention due to rheumatic lesions, since this condition is associated with the high perioperative and long-term mortality and morbidity^19–22^. Although studies have associated high PLA with vascular inflammation-associated diseases such as angina and stroke, the present prospective study appears to be the first focusing on preoperative PLA levels and MACCE after cardiac surgery.

## Results

Baseline characteristics of the patients are shown in Table 1. Nearly one-third (32%) were men, mean age was 47 years old, and most (84.8%) were NYHA class III. Median stay in the intensive care unit was 46 hours (IQR 41–69 hours); mean hospital stay was 9 ± 3 days.

**Table 1:** Perioperative features of patients*.

Feature	Total (n = 244)	PLA		*p* value
		Low (≤6.8%) (n = 93)	High (>6.8%) (n = 151)	
**Demographics**				
Age, yr	47 ± 9	47 ± 9	48 ± 9	0.500
Male, n (%)	78 (32.0)	29 (31.2)	49 (32.5)	0.837
Body mass index, kg/m^2^	22.3 ± 2.8	22.3 ± 2.9	22.2 ± 2.7	0.771
Smoking, n (%)	52 (21.3)	19 (20.4)	33 (21.9)	0.792
**Medical history, n (%)**				
New York Heart Association functional class				0.486
II	37 (15.2)	16 (17.2)	21 (13.9)	
III	207 (84.8)	77 (82.8)	130 (86.1)	
Diabetes	5 (2.0)	1 (1.1)	4 (2.6)	0.706
Atrial fibrillation	118 (48.4)	40 (43.0)	78 (51.7)	0.189
Hypertension	18 (7.4)	8 (8.6)	10 (6.6)	0.566
Left atrial thrombus	33 (13.5)	9 (9.7)	24 (15.9)	0.168
**Type of valvular disease, n (%)**				
Mitral valve				0.354
Stenosis	71 (29.1)	22 (24.4)	49 (32.9)	
Regurgitation	35 (14.3)	14 (15.6)	21 (14.1)	
Stenosis and regurgitation	105 (43.0)	40 (44.4)	65 (43.6)	
Aortic valve				0.980
Stenosis	9 (3.7)	3 (3.3)	6 (4.0)	
Regurgitation	86 (35.2)	33 (36.7)	53 (35.6)	
Stenosis and regurgitation	75 (30.7)	29 (32.2)	46 (30.9)	
Tricuspid regurgitation	116 (47.5)	39 (43.3)	77 (51.7)	0.211
**Echocardiographic data**				
Left ventricle ejection fraction, %	60 ± 13	60 ± 14	61 ± 12	0.506
Left ventricle diameter, mm	51 ± 12	52 ± 12	50 ± 11	0.086
Left atrial diameter, mm	51 ± 17	49 ± 17	52 ± 17	0.271
Right ventricle diameter, mm	21 ± 5	21 ± 6	21 ± 5	0.629
EuroSCORE, n (%)				0.118
Low (0–2)	227 (93.0)	89 (95.7)	138 (91.4)	
Medium (3–5)	16 (6.6)	3 (3.2)	13 (8.6)	
High (≥6)	1 (0.4)	1 (1.1)	0 (0.0)	
**Medications, n (%)**				
Warfarin	3 (1.2)	1 (1.1)	2 (1.3)	>0.999
Aspirin	11(4.5)	4 (4.3)	7 (4.6)	>0.999
Calcium antagonists	2 (0.8)	0 (0.0)	2 (1.3)	0.526
β-blocker	19 (7.8)	6 (6.5)	13 (8.6)	0.541
Digoxin	26 (10.7)	12 (12.9)	14 (9.3)	0.372
Insulin	1 (0.4)	1 (1.1)	0 (0.0)	0.381
Angiotensin-converting enzyme inhibitor	9 (3.7)	4 (4.3)	5 (3.3)	0.961
Diuretics	26 (10.7)	11 (11.8)	15 (9.9)	0.641
**Blood cell count before surgery**				
Leukocytes (×10^9^/L)	4.69 ± 1.60	4.66 ± 1.42	4.70 ± 1.71	0.857
Platelets (×10^9^/L)	137 ± 48	128 ± 50	143 ± 46	0.020
Red blood cells (×10^12^/L)	4.25 ± 0.56	4.17 ± 0.53	4.29 ± 0.57	0.133
**In operating room**				
Valve replaced, n (%)				0.297
Aortic	41 (16.8)	20 (21.7)	21 (14.0)	
Mitral	98 (40.2)	35 (38.0)	63 (42.0)	
Aortic and mitral	103 (42.2)	37 (40.2)	66 (44.0)	
Bioprosthetic valves, n (%)	16 (6.6)	7 (7.5)	9 (6.0)	0.631
Concomitant tricuspid repair, n (%)	112 (45.9)	41 (44.1)	71 (47.0)	0.655
Maze, n (%)	65 (26.6)	17 (18.3)	48 (31.8)	0.020
CPB time, min	117 ± 36	122 ± 38	115 ± 34	0.131
Cross-clamp time, min	79 ± 30	80 ± 30	78 ± 30	0.663
Packed red blood cell consumption, units	0 (0,1.50)	0 (0,1.50)	0 (0,1.50)	0.993
MACCE	30 (12.3)	5 (5.5)	25 (17.1)	0.009
Intensive care unit stay, hours	46 (41, 69)	46 (41, 69)	46 (42, 69)	0. 951
Hospital stay, days	10 ± 3	10 ± 3	9 ± 3	0.015

CPB, cardiopulmonary bypass; MACCE, major adverse cardiac and cerebrovascular events.

^#^A total of 244 patients were included.

### PLA and baseline variables

Median PLA was 9.6% (IQR 4.5–27.0%). Median TNF-α was 1.4 pg/ml (IQR 0.8–2.3 pg/ml), which did not correlate with PLA (r = −0.093, *p* = 0.159). Platelet count was 137 × 10^9^/L, and it positively correlated with PLA (r = 0.187, p = 0.003, Spearman’s rank). In univariate analysis, higher preoperative platelet count was associated with higher PLA (unadjusted OR 1.01, 95% CI 1.00–1.01; p = 0.022).

We explored potential correlations of elevated PLA with pre- and perioperative characteristics. We found that elevated PLA correlated positively with platelet count before surgery (r = 0.129, p = 0.04), but not with leukocyte count (r = 0.025, p = 0.70). However, no other significant associations were identified between elevated PLA and pre- or perioperative characteristics, including age (r = −0.040, p = 0.54), gender (r = −0.503, p = 0.41) or BMI (r = 0.039, p = 0.54), or comorbidities such as hypertension (r = −0.036, p = 0.57), diabetes (r = −0.001 p = 0.99), or atrial fibrillation (r = 0.020, p = 0.75).

### Incidence of MACCE and perioperative AEs

Primary outcome was major cardiac and cerebrovascular events (MACCE), a composite index consisting of stroke, heart failure, myocardial infarction, life-threatening arrhythmia, transient ischemic attack and MACCE-related death. A total of 13 (5.48%) patients died during the 3-year follow-up, and 8 deaths could be attributed to MACCE: 6 to cardiac failure, 1 to stroke and 1 to sudden death. The remaining 5 deaths were due to gastric cancer (n = 2), traffic accidents (n = 2) or lung infection (n = 1).

During follow-up, 30 patients (12.7%) developed MACCE, which comprised MACCE-related death (n = 8), heart failure progression (n = 6), stroke (n = 8), life-threatening arrhythmia (n = 6), transient ischemic attack (n = 1), as well as myocardial infarction (n = 1) (Table 2). Multivariate Cox regression identified no risk factors for perioperative AEs (Supplementary Table 4).

**Table 2:** Distribution of MACCE and perioperative adverse events.

	n (%)
**All MACCE**	**30** (**12.7)**
MACCE-related death	8 (3.4)
Heart failure progression	6 (2.5)
Stroke	8 (3.4)
Life-threatening arrhythmia	6 (2.5)
Transient ischemic attack	1 (0.4)
Myocardial infarction	1 (0.4)
**Perioperative adverse events**	38 (15.6)
Respiratory failure	18 (7.4)
Acute kidney injury	15 (6.1)
Neurological complications	7 (2.9)
Cardiac adverse events	6 (2.5)

MACCE, major adverse cardiac and cerebrovascular events.

Secondary outcome was perioperative adverse events (AEs), defined as cardiac AEs, acute kidney injury, neurological complications and respiratory failure from the date of surgery until 30 days after surgery. Perioperative AEs occurred in 38 patients (15.6%), 8 of whom had 2 types of AEs. The total inventory of these AEs was respiratory failure (n = 18), acute kidney injury (n = 15), neurological complications (n = 7) and cardiac AEs (n = 6) (Table 2). Patients with perioperative AEs were more likely to be smoker than those without perioperative AEs (34.2% vs 18.9%; *p* = 0.035), and had higher PLA (23.55% vs 9.01%; *p* = 0.004). The two groups had similar TNF-α (1.50 pg/ml vs 1.46 pg/ml; p = 0.865). Multivariate logistic regression identified aortic valve replacement, concomitant tricuspid repair and increased PLA (adjusted OR 1.24, 95% CI 1.10–1.39; p = 0.001) as risk factors for perioperative AEs (Supplementary Table 3).

### PLA and outcomes

**Figure 1:**
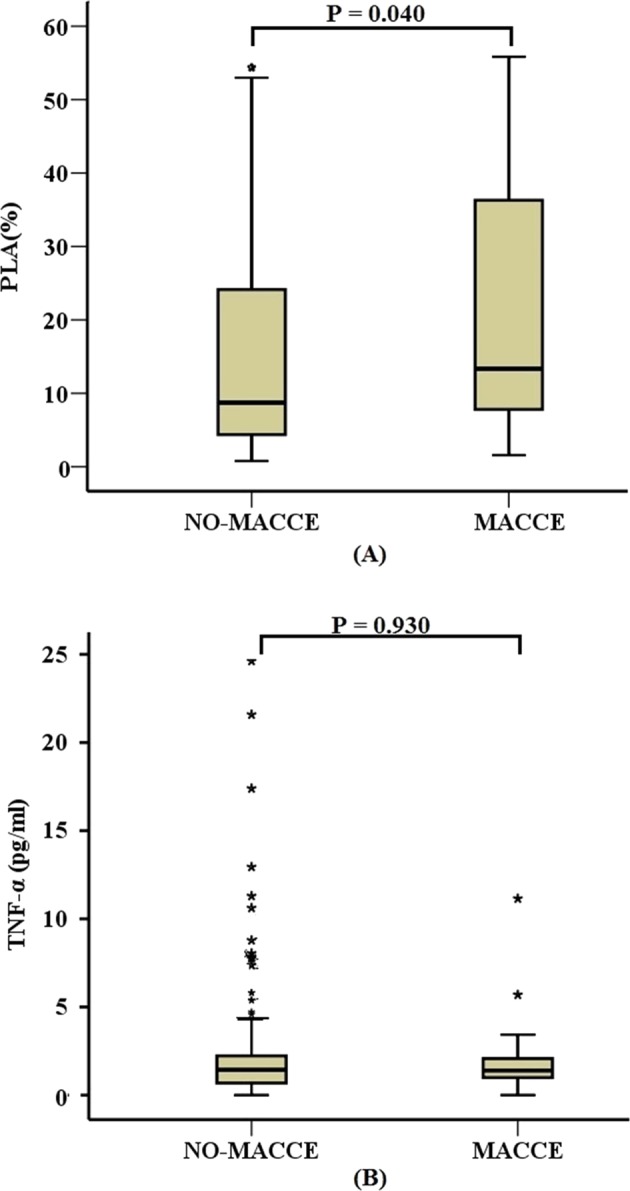
PLA and TNF-α level in patients with or without MACCE. (**A**) Patients with MACCE had higher PLA level than those without MACCE (13.32% vs 8.69%; *P* = 0.040). (**B**) Patients with MACCE and patients without MACCE had similar TNF-α level (1.40 pg/ml vs 1.43 pg/ml; *p* = 0.930). Differences were tested for significance using the rank sum test. The middle horizontal line indicates the median; the upper whisker, the maximum non-outlier; the lower whisker, the minimum non-outlier; and extra dots, outliers. Data are shown for 237 patients who completed 3-year follow-up.

Patients who developed MACCE had higher PLA than those who did not (13.32% vs 8.69%; P = 0.040; Fig. 1A). Multivariate Cox regression indicated that the occurrence of MACCE correlated significantly with PLA for each 5% increase (Table 3). When patients were divided into quartiles based on PLA (Group 1, ≤4.5%; Group 2, from 4.5% to 9.6%; Group 3, from 9.6% to 27%; and Group 4, >27%; n = 61 in each group), increasing PLA was found to be associated with both MACCE (Table 3) and perioperative AEs (Table 4) in a step-wise manner.

**Table 3:** Results of unadjusted and adjusted Cox regression analysis of MACCE.

PLA comparison	Unadjusted	p	Adjusted #	p
	HR (95% CI)		HR (95% CI)	
Each 5% increase	1.11 (0.99, 1.24)	0.067	1.13 (1.01, 1.26)	0.037
Each quartile	1.46 (1.04, 2.05)	0.028	1.51 (1.07, 2.13)	0.020
PLA ≤ 6.8% vs. PLA > 6.8%	3.43 (1.31, 8.96)	0.012	3.49 (1.33, 9.11)	0.011

^#^Adjusted by age, gender, body mass index, smoking history, NYHA functional class, diabetes, stroke history, atrial fibrillation, hypertension, digoxin, types of valve replacement and concomitant tricuspid repair.

**Table 4:** Results of unadjusted and adjusted logistic regression analysis of perioperative AEs.

PLA comparison	Unadjusted	p	Adjusted #	p
	OR (95% CI)		OR (95% CI)	
Each 5% increase	1.19 (1.06, 1.32)	0.002	1.24 (1.10, 1.39)	0.001
Each quartile	1.51(1.09,2.10)	0.013	1.61 (1.14,2.26)	0.007
PLA ≤ 6.8% vs. PLA > 6.8%	2.63 (1.15, 6.03)	0.022	3.01 (1.27, 7.12)	0.012

^#^Adjusted by confounding factors and CPB time. age, gender, body mass index, smoking history, NYHA functional class, diabetes, stroke history, atrial fibrillation, hypertension, digoxin, types of valve replacement and concomitant tricuspid repair, CPB time.

**Figure 2:**
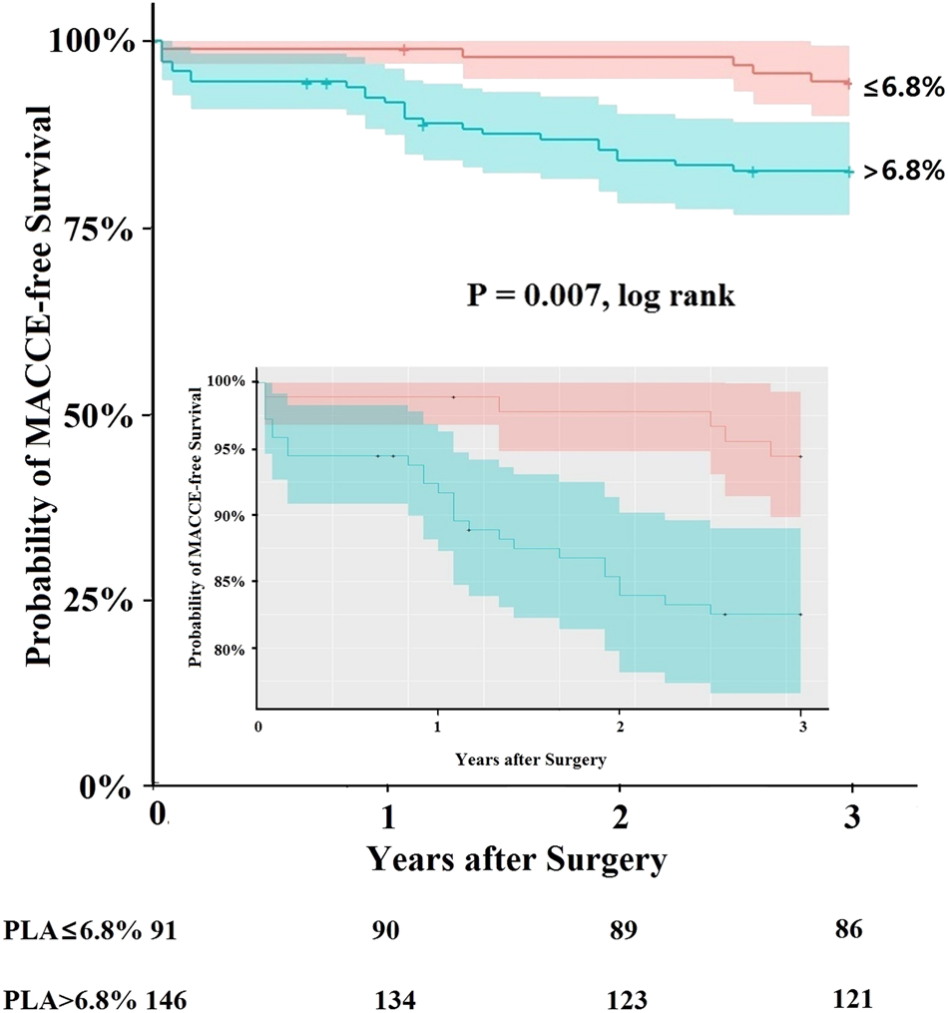
Influence of PLA on MACCE-free survival. Kaplan-Meier analysis of 3-year MACCE-free survival among patients with ≤6.8% PLA and patients with PLA > 6.8%. The inset shows the same plot with an adjusted y-axis to highlight differences between the patient groups.

ROC curve analysis identified the optimal PLA cut-off to be 6.8% with sensitivity of 0.833, specificity of 0.415, and an area under the curve of 0.616 (95% CI 0.515 to 0.718). Rate of MACCE was significantly higher in patients with >6.8% PLA (25/146, 17.1%) than in patients with ≤6.8% PLA (5/91, 5.5%; p = 0.009). In patients with >6.8% PLA, adjusted HR for MACCE was 3.49 (95% CI 1.33 to 9.11; p = 0.011) (Table 3), and MACCE-free survival was shorter than for patients with ≤6.8% PLA, based on Kaplan-Meier analysis (p = 0.007, log rank; Fig. 2). Patients with >6.8% PLA also had higher rates of perioperative AEs (19.9% vs 8.6%, *p* = 0.018). Logistic analyses revealed an association of increasing PLA with increasing perioperative AEs (Table 4).

Incidences of MACCE (7.1% vs 13.7%, *p* = 0.491) and perioperative AEs (21.4% vs 17%, *p* = 0.970) were comparable between patients who received or did not receive anticoagulation therapy (aspirin and/or warfarin) prior to surgery. The HR of MACCE for PLA > 6.8% was not significantly altered after adjusting for use of anticoagulation therapy (adjusted HR 3.44, 95% CI 1.32–8.99; p = 0.012).

### TNF-α and outcomes

Patients who developed MACCE had similar TNF-α (1.40 pg/ml vs 1.43 pg/ml; p = 0.930; Fig. 1B). TNF-α was not associated with MACCE or perioperative AEs (Supplementary Table 2).

## Discussion

In this prospective cohort study, we found that the incidence of MACCE was 4.2% per year in patients after valve surgery, indicating these patients may be at high risk of cardiovascular morbidity. Elevated PLA prior to surgery was associated with increased rate of perioperative AEs and MACCE during a 3-year period. Patients with preoperative PLA > 6.8% were more likely to suffer these outcomes. These results suggest that PLA could be used as a biomarker to identify patients with rheumatic valve disease at high risk of MACCE after surgical intervention. This is, to our knowledge, the first report suggesting an association of preoperative PLA with risk of perioperative AEs and MACCE.

Previous studies^23–28^ have identified a variety of biomarkers that can predict morbidity and mortality after cardiac surgery. For example, postoperative B-type natriuretic peptide at >790 ng/L and troponin T at >0.08 μg/L have been associated with mid- and long-term mortality and major cardiac events after cardiac surgery. High preoperative C-reactive protein has also been associated with higher rate of mortality, cardiovascular events and myocardial damage after cardiac surgery. However, elevation of these biomarkers is the result, not cause, of tissue damage.

Both innate and adaptive immune cells are activated in inflammation-associated vascular lesions^5,29^. CD62L, an adhesion molecule expressed on lymphocytes, monocytes and neutrophils^5,15,30^, was used to identify leukocytes in the present study. CD62L has been shown to be up-regulated in stroke patients^15^, and to cluster along the leading edge of leukocytes during platelet-leukocyte interaction^30^. Lymphocytes from CD62L^−/−^ animals have impaired ability to migrate to atherosclerotic aortas^5,31^. These results suggest that CD62L contributes directly to vascular lesions.

Based on double staining with CD62L and CD41a, the median PLA level in the current study was 9.6%, which was more than double the rate reported for a healthy population (3.5%)^32^. Increased PLA may induce and amplify inflammation via release of pro-coagulatory^33^ and pro-inflammatory^34,35^ factors. For this reason, increased PLA might partly explain the phenomenon that patients with rheumatic disease are at high risk of coronary artery disease (CAD) events^36^. Increased PLA can also be seen in patients with ischemic and non-ischemic cardiac and cerebrovascular events, such as angina^37^, acute coronary syndrome^12,13,16,38^, and cerebrovascular ischemia^15,17,39^. If increased PLA is a risk factor of CAD, it may also increase risk of MACCE by inducing similar cerebral vascular inflammation. Consistent with this, our results demonstrate an association of increased PLA with increased MACCE and perioperative AEs, and previous reports have linked increased PLA with stroke^15,32^ and angina^13,16^.

In patients with rheumatic disease, levels of the systemic inflammatory factor TNF-α play an important role in occurrence and development of disease^40^. However, we found in the present study that TNF-α did not correlate significantly with either MACCE or perioperative AEs. This may mean that TNF-α is not a reliable marker of vascular inflammation, despite its role in regulating immune cell activation^9^. This apparent paradox should not be surprising, given the complexity of the cytokine and chemokine networks involved.

Our results, together with others, suggest PLA may be more effective than TNF-α to predict MACCE in patients with rheumatic valve disease, which may be due to the role of PLA in cardiovascular lesions. Cardiac and cerebrovascular injury activate inflammatory and coagulation responses, as well as the corresponding antagonistic pathways. The interaction between platelets and leukocytes leads to leukocyte accumulation in the vascular wall, myocardium and many other organs. These PLA adhere to the endothelium at sites prone to plaque formation^11^. Leukocyte infiltration into tissue can promote collagen breakdown and structural remodeling processes, triggering oxidative stress^41^, leading in turn to ischemic heart failure^42^. Infiltrating leukocytes and PLA seem to promote structural and electrical remodeling in patients with atrial fibrillation^43^, which may also result in heart dysfunction and even failure.

To further elucidate the predictive value of PLA on MACCE, we used ROC analysis to identify an optimal PLA cut-off of 6.8% for stratifying our study population based on risk of MACCE. This level is similar to that previously reported in patients with cardiac and cerebrovascular ischemia, and lower than that reported in ischemic stroke patients (8.9%)^32^, and approximately twice the value reported in a healthy population (3.5%)^32^. These results suggest that the cellular biomarker PLA may predict higher risk of adverse cardiovascular events, perhaps because high PLA reflects a chronic proinflammatory state.

Some studies have reported that PLA correlates with platelet count and P-selectin, but not with white cell count or neutrophil activation (L-selectin)^44^. We found that elevated PLA correlated positively with platelet count before surgery but not with leukocyte count. No other significant associations were identified between increased PLA and pre- or perioperative characteristics.

The current study was conducted in a single center with a relatively small sample, 84% of them with poor cardiac function (NYHA III-IV), which may create bias, although the NYHA class was adjusted in logistic analysis. Second, although we composited small events (from 1 to 18) to MACCE and AEs, according to the similar studies^45–50^, there still a chance to create bias in analysis the PLA and the outcomes. At last, we did not attempt to identify which subtype(s) of leukocytes in PLA may explain our results. Monocytes, as macrophage precursors, migrate early to the endothelium^8^, while T helper 1 lymphocytes secrete cytokines to activate macrophages^51^, and infiltrating neutrophils help enlarge and destabilize atherosclerotic lesions by creating oxidative stress^8^. Future work should examine the contributions of these different subtypes to MACCE.

### Clinical implications

Our findings suggest that PLA can serve as a useful cellular biomarker of MACCE risk in patients undergoing rheumatic valve replacement. It may be possible to monitor PLA in order to stratify patients according to MACCE risk, and it may be useful as a treatment target to reduce such risk. Indeed, animal studies^18,52^ have proven that blocking PLA formation can effectively attenuate vascular lesions, although how this occurs requires further study. It is possible that PLA is equally useful for patients undergoing other types of cardiac surgery, such as CABG, which should be explored in future work. Our results suggest that future clinical and laboratory studies should focus on PLA as a cellular biomarker of MACCE risk and on the role of PLA in formation and progression of vascular lesions. We highlight the need for further research to identify preoperative variables that affect PLA levels. It is possible that inhibiting PLA formation via the preoperative variables might improve the patient prognosis, and this should be explored in larger samples.

## Methods

### Patients

**Figure 3:**
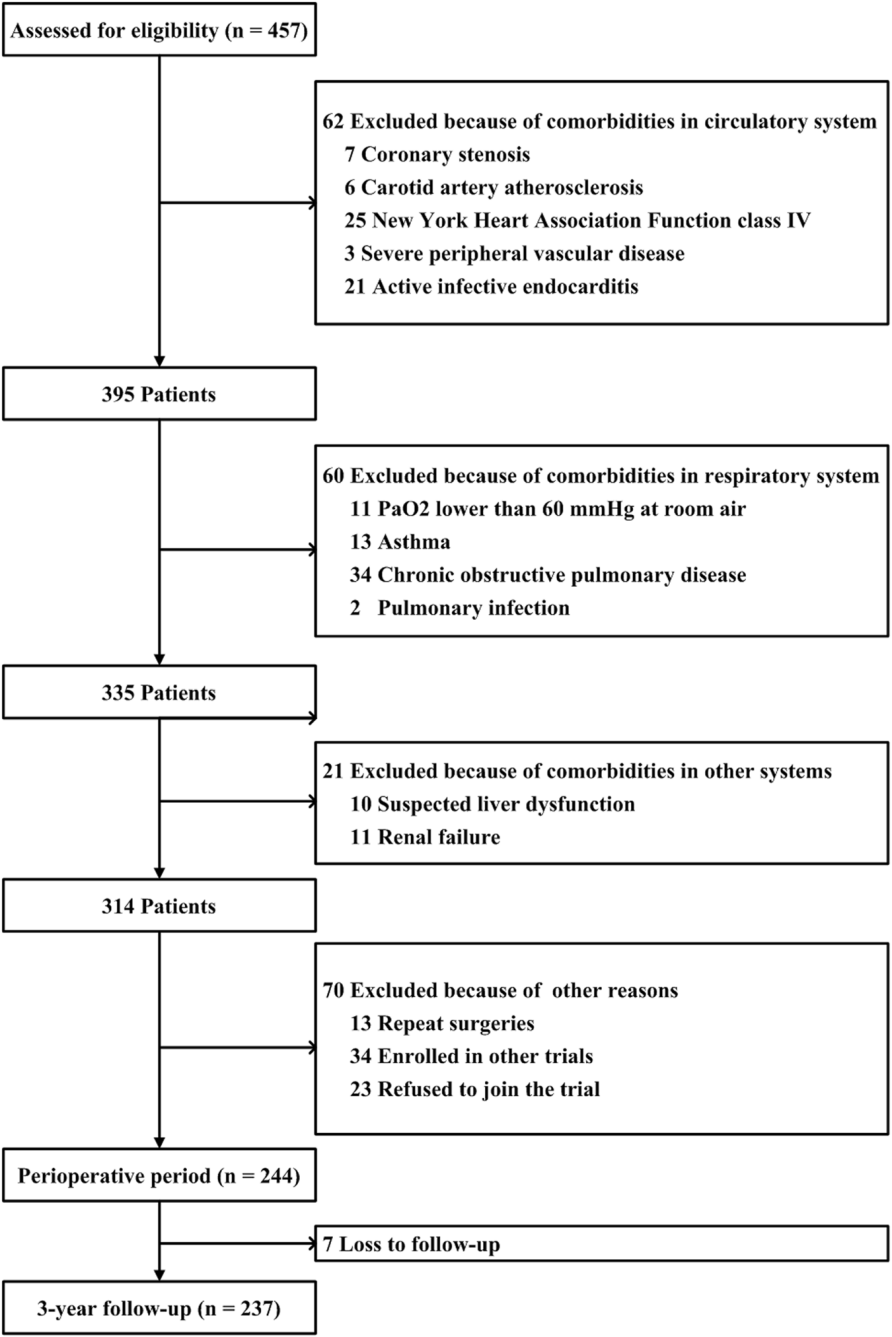
Flowchart of patient selection and enrollment.

This prospective cohort included adult patients (18–65 years old) with rheumatic valve disease who underwent surgical intervention at West China Hospital, Sichuan University between November 1, 2011 and September 30, 2012. Of the 457 potentially eligible patients, 62 were excluded because of preoperative complications in the circulatory system: coronary stenosis (n = 7), carotid artery atherosclerosis (n = 6), NYHA class IV status (n = 25), severe peripheral vascular disease (n = 3) or active infective endocarditis (n = 21). Another 60 patients were excluded because of preoperative complications in the respiratory system: PaO2 lower than 60 mmHg with room air (n = 11), asthma (n = 13), chronic obstructive pulmonary disease (n = 34) or pulmonary infection (n = 2). Another 21 were excluded because of a history of suspected liver injury (n = 10) or renal failure (n = 11). A further 70 patients were excluded for the following reasons: repeated surgery (n = 13), enrollment in other clinical trials (n = 34), or refusal to join the trial (n = 23). In the end, analysis of perioperative AEs included 244 subjects, while 7 patients (2.87%) were lost to follow-up and so only the remaining 237 patients were included in analysis of MACCE (Fig. 3). All 237 patients completed 3-year follow-up.

All subjects signed written informed consent. The study protocol was approved by the Ethics Committee of Sichuan University and conducted in accordance with the Declaration of Helsinki. The study was registered in the Chinese Clinical Trial Registry (ChiCTR-OCH-12001922).

### Patient management and procedure

Patients managements, including anesthesia, cardiopulmonary bypass (CPB), were performed according to standard hospital protocols (Supplementary Information 3)^53^.

All patients received postoperative warfarin, but not anti-platelet agents, as anticoagulation strategy. The target international normalized ratio (INR) in the present study was 1.5–2.5 for mechanical mitral valve replacement, 1.3–1.8 for mechanical aortic valve replacement; and 2.0–3.0 for tricuspid valve replacement, which is lower than the INR recommended in a Western study^54^. The lower INR is routinely used at our hospital and many medical centers in China because it appears to be more appropriate for Asian populations based on several studies^55–57^.

### Assessment of PLA and TNF-α

Blood samples for PLA and TNF-α were collected after anesthesia induction. Samples were incubated with CD62L-PE (BD, Basel, Switzerland), CD41a-FITC (BD) and DRAQ5 (Biotium, Hayward, CA, USA). An IgG1-FITC/PE antibody (BD) served as an isotype control. PLA was identified using CD62L/CD41a dot plots after confirming cell nuclei using DRAQ5. Putative PLA was verified by sorting stained samples using FACSAria and observation under a confocal fluorescence microscope (Supplementary Figure). Details of PLA determination are provided in Supplementary Information 4.

For analysis of plasma TNF-α, arterial blood was centrifuged at 4 °C for 15 min at 1000 *g*. TNF-α concentration was determined using a commercial enzyme-linked immunosorbent assay kit (High Sensitivity Kit, R&D Systems, Minneapolis, MN, USA) with a detection limit of 0.038 pg/ml.

### Data collection

Baseline variables included demographics, smoking history, cardiac disease, NYHA class, history of medication, preoperative comorbidities and preoperative blood cell counts. Intraoperative variables included type of valve replacement, CPB time, cross-clamp time and blood transfusion.

Follow-up was carried out every 6 months for three years, either when patients came into hospital for treatment or by telephone. Patients or their family members were asked to report whether and when the patients had been hospitalized in any other hospitals. If the patients were hospitalized in our hospital, study events were determined during their hospitalization. For the other re-admission patients, events were determined based on medical records, and, when necessary, discussion with the consulting physician. Researchers conducting follow-up were blinded to patients’ PLA results.

### Outcomes

Primary outcome was MACCE, including stroke, heart failure, myocardial infarction, life-threatening arrhythmia, transient ischemic attack and MACCE-related death. Re-hospitalizations for a combination of heart failure and arrhythmia were counted only once, and the reason was recorded as heart failure if heart failure persisted after treatment of arrhythmia; otherwise, the reason was recorded as arrhythmia.

Secondary outcome was AEs. Detailed definitions of the AEs are listed in *Supplementary definition of outcomes*.

### Statistical analysis

The comparison of baseline characteristics was used to select potential risk and confounding factors. All variables, including demographics, medical history, preoperative medication, blood cell counts and intraoperative variables, were included into univariate and multivariate backward Cox proportional hazard regression to assess their association with 3-year outcomes. Logistic regression was used to access the association between variables and perioperative outcomes. To identify the effect of PLA on outcomes, hazard ratio (HR) was adjusted by age, gender, body mass index, smoking history, NYHA functional class, diabetes, stroke history, atrial fibrillation, hypertension, digoxin, type of valve replacement, concomitant tricuspid repair and valve material. Odds ratio (OR) was adjusted by all these factors as well as CPB time.

We performed receiver operating characteristic (ROC) curves, based on MACCE and PLA, from the logistic regression model, and then Youden’s index were used to identify the optimal PLA cut-off value for predicting MACCE in 3 years. This threshold was used to classify patients as having low or high PLA, and MACCE-free survival was compared between the two groups using Kaplan-Meier analysis and the log rank test. Our pilot results with 88 patients showed that 2 of 34 patients with PLA ≤ 6.8% suffered MACCE, compared to 9 of 54 patients with PLA > 6.8%. The sample size was calculated by Cox model based on 2-Sided Equality, according to the Time-To-Event data. Power analysis indicated the need for at least 193 patients, based on 1-β = 0.8, α = 10%. Based on the number of cardiac surgery patients at our hospital in 2010 and the exclusion criteria in our study, we decided to screen all valve replacement patients within one year for eligibility.

All statistical analyses were performed using SPSS 19.0 (IBM, Chicago, IL, USA) and RStudio 1.1.453 (R-Tools Technology, Canada). In all analyses, p < 0.05 was considered significant. Detailed methods of the statistical analysis are shown in *Supplementary statistical analysis*.

## Supplementary information

Supplemental information

## Data Availability

The datasets used and/or analyzed during the current study are available from the corresponding author on reasonable request.
