# Supplementary material for: Platelet-leukocyte aggregate is associated with adverse events after surgical intervention for rheumatic heart disease
Source: Sci Rep. 2019 Sep 10;9:13069. doi: 10.1038/s41598-019-49253-3 (PMC6737193; doi:10.1038/s41598-019-49253-3)
Supplement: Supplementary file 1 — Supplemental information [file 41598_2019_49253_MOESM1_ESM.docx]

**Title Page**

**Platelet-leukocyte aggregate is associated with adverse events after surgical intervention for rheumatic heart disease**

Chaonan Liu, MD ^1,^*,Yang Yang, MD ^2,^*^,&^, Lei Du, PhD ^2,^*, Si Chen, MD ^1^, Jie Zhang, MD ^3^, Chongwei Zhang, MD ^1^, Jing Zhou, MD ^1,#^

^1^ Department of Laboratory Medicine, West China Hospital, Sichuan University, Chengdu, China

^2^ Department of Anesthesiology, West China Hospital, Sichuan University, Chengdu, China.

^3^ Key Laboratory of Transplant Engineering and Immunology, West China Hospital, Sichuan University, Chengdu, China.

* Liu C, Yang Y and Du L contributed equally to this work.

^&^ Current Address: Department of Anesthesiology, Affiliated Hospital of Guiyang Medical College

^#^ Correspondence to Jing Zhou, MD, Department of Laboratory Medicine, West China Hospital, Sichuan University, No. 37, Guoxuexiang, Chengdu, Sichuan, 610041 China. Phone and Fax: +86-28-8542-2613. E-mail address: [zhoujinghuaxi@163.com](mailto:zhoujinghuaxi@163.com).

**Table of contents**

| Supplementary Information 1 | Definition of comorbidities |
| --- | --- |
| Supplementary Information 2 | Definition of outcomes |
| Supplementary Information 3 | Supplementary methods of patient management |
| Supplementary Information 4 | Assessment of PLA |
| Supplementary Information 5 | Supplementary statistical analysis |
| Supplementary Information 6 | Supplementary Figure |
| Supplementary Information 7 | Supplementary Tables |
| Supplementary Information 8 | Supplementary References |

**Supplementary Information 1**

**Definition of comorbidities**

**Infective endocarditis**^1^(IE) was defined to meet two major clinical criteria, or one major and three minor criteria, or five minor criteria:

Major criteria included:

1. Positive blood culture with a typical IE microorganism, defined as one of the

following:

- Typical microorganism consistent with IE from two independent blood

cultures, as noted below:

- *Viridans*-group streptococci, or

- *S. bovis* including nutritional variant strains, or

- HACEK group, or

- *S. aureus,* or

- Community-acquired enterococci, in the absence of a primary focus

- Microorganisms consistent with IE from persistently positive blood cultures

defined as:

- Two positive cultures of blood samples drawn >12 hours apart, or

- all of 3 or 3 of 4 separate cultures of blood (with the first and last

samples drawn 1 h apart)

- *Coxiella burnetii* detected by at least one positive blood culture or antiphase I IgG antibody titer >1:800

2. Evidence of endocardial involvement, with a positive echocardiogram defined as

- Oscillating intracardiac mass on valve or supporting structures, in the path of

regurgitant jets, or on implanted material in the absence of an alternative

anatomical explanation, or

- Abscess, or

- New partial dehiscence of prosthetic valve or new valvular regurgitation

(worsening or changing of preexisting murmur insufficient)

Minor criteria included:

1. Predisposing factors: known cardiac lesion, recreational drug injection

2. Fever >38 °C

3. Evidence of embolism: arterial emboli, pulmonary infarcts, Janeway lesions,

conjunctival hemorrhage

4. Immunological problems: glomerulonephritis, Osler's nodes

5. Positive blood culture not meeting a major criterion, or serological evidence of

infection with an organism consistent with IE without satisfying a major criterion

**Severeperipheralarterydisease**^2^ was defined as rest pain, ulceration or gangrene, atheroembolism of lower extremities, and thrombosis of abdominal aorta.

**Active infective endocarditis**^3^ was defined as:

1. infective endocarditis with persistent fever and positive blood cultures, or

2. active inflammatory morphology found at surgery, or

3. on-going antibiotic therapy, or

4. histopathological evidence of active infective endocarditis

**Asthma**^4^was diagnosed if the patient required,during the previous year,treatment with high-dose inhaled corticosteroids and long-acting β2-agonists or leukotriene modifier/theophyllinebased on Global Initiative for Asthma(GINA) guidelines for step 4-5 asthma, or systemic corticosteroids for more than half of the previous year to prevent the condition from becoming ‘‘uncontrolled’’. Cases in which the condition remained ‘‘uncontrolled” despite this therapy were also included.

Uncontrolled asthma was defined as at least one of the following:

1) Poor symptom control: Asthma Control Questionnaire consistently >1.5, Asthma

Control Test >20 or disease rated as ‘‘not well controlled’’ based on guidelines of

GINA or the National Asthma Education and Prevention Program.

2) Frequent severe exacerbations: two or more bursts of systemic corticosteroids (>3

days each) in the previous year

3) Serious exacerbations: at least one hospitalization, ICU stay or mechanical ventilation

in the previous year

4) Airflow limitation as established by FEV1 <80% predicted after appropriate

bronchodilation (this threshold was defined as below the lower normal limit in the

presence of reduced FEV1/FVC)

Controlled asthma was defined as asthma that worsened on tapering of high-dose

inhaled corticosteroids, systemic corticosteroids or additional biologics.

**Chronic obstructive pulmonary disease**^5^ was defined as incompletely reversible airway obstruction, which was defined as a ratio of post-bronchodilator forced expiratory volume in 1 s to forced vital capacity (FEV1:FVC) less than 70%.

**Pulmonary infection**^6^was defined as when a patient was on antibiotic therapy and presented one of the following: radiographic evidence of new or changed lung opacity, a change in color or consistency of sputum, or fever.

**Suspected liver dysfunction**^7^ was defined as an increase of over two times the upper limit of the normal range (2N) in serum alanine aminotransferase (ALT) or conjugated bilirubin or a combined increase of aspartate aminotransferase (AST), alkaline phosphatase (AP), and total bilirubin, provided one of them was above 2N.

**Renal failure**^8^ was defined as a glomerular filtration rate (GFR) <15ml/min/1.73m^3^.

**Supplementary Information 2**

**Definition of outcomes**

An **ischemic stroke**^1^ was an acute episode of focal or global neurological dysfunction caused by brain, spinal cord, or retinal vascular injury as a result of infarction of central nervous system tissue. Hemorrhage could be a consequence of ischemic stroke; in this situation, the stroke was defined as ischemic (not hemorrhagic) with hemorrhagic transformation.

**Hemorrhagic stroke**^1^ was defined as an acute episode of focal or global cerebral or spinal dysfunction caused by intraparenchymal, intraventricular, or subarachnoid hemorrhage.

**Undetermined stroke**^1^ was defined as an acute episode of focal or global neurological dysfunction caused by presumed brain, spinal cord, or retinal vascular injury as a result of hemorrhage or infarction, for which insufficient information was available to allow categorization as ischemic or hemorrhagic.

**Heart failure**^9^was defined as a complex clinical syndrome resulting from any structural or functional cardiac disorder that impairs the ability of the ventricle to fill with, or eject, blood.

**Myocardial infarction**^1^(MI) was defined as an acute ischemic event associated with documented and clinically significant myocardial necrosis. MI was periprocedural (≤72 h after the procedure) or spontaneous (>72 h after the index procedure).

1. *Peri-procedural MI*satisfiedboth of the following criteria:

(a) new ischemic symptoms (e.g., chest pain or shortness of breath), or new ischemic signs (e.g. ventricular arrhythmias, new or worsening heart failure, new ST-segment changes, hemodynamic instability, or imaging evidence of new loss of viable myocardium or new wall motion abnormality); and

(b) elevated cardiac biomarkers (preferably CK-MB) within 72 h after the index procedure, consisting of two or more post-procedure samples that were > 6-8 h apart with a 20% increase in the second sample and a peak value exceeding 10 times the 99th percentile of the upper limit of the reference value (URL), or a peak value exceeding 5 times the 99th percentile URL with new pathological Q waves in at least 2 contiguous leads.

2. *Spontaneous MI*satisfiedone of the following criteria:

(a) Rise and/or fall in cardiac biomarkers (preferably troponin) with at least one value above the 99th percentile URL, together with evidence of myocardial ischemia with at least one of the following: (1) ECG changes indicative of new ischemia (new ST-T changes or new LBBB); (2) new pathological Q waves in at least two contiguous leads; (3) imaging evidence of new loss of viable myocardium or new wall motion abnormality.

(b) Sudden, unexpected cardiac death involving cardiac arrest, often with symptoms suggestive of myocardial ischemia, and accompanied by presumably new ST-segment elevation, or new LBBB, and/or evidence of fresh thrombus based on coronary angiography and/or autopsy, but death occurring before blood samples couldbe obtained, or before the appearance of cardiac biomarkers in the blood.

(c) Pathological findings of an acute myocardial infarction.

**Life-threatening arrhythmia**^10^was defined as ventricular tachycardia lasting >30 sec, resuscitated cardiac arrest, or appropriate implantable cardioverter defibrillator (ICD) therapy (antitachycardia pacing or defibrillation).

A **transient ischemic attack**^1^ (TIA) was a transient episode of neurological dysfunction caused by focal brain, spinal cord, or retinal ischemia, without acute infarction.

**Acute kidney injury**^11^was defined as an increase in serum creatinine of ≥0.3 mg/dl (≥26.4 μmol/L) within 48 h or an increase in serum creatinine to 1.5 times baseline.

**Neurological complications**^12^were defined as a broad spectrum of complications including seizures, coma, cerebral hemorrhage or stroke during hospitalization.

**Respiratory failure**^13^was diagnosed if patients had hypoxemia and/or hypercapnia, i.e. arterial oxygen pressure <60 mmHg and/or arterial carbon dioxide pressure >50 mmHg while breathing air.

**Supplementary Information 3**

**Supplementary methods of patient management**

Anesthesia was induced with fentanyl supplemented with midazolam and non-depolarizing muscle relaxants and maintained with propofol and/or inhalation anesthetics. Central venous pressure (CVP), direct arterial pressure, pulse oxygen saturation, and end-tidal CO_2_ were continuously monitored throughout surgery. Arterial blood gas was determined when necessary. All surgical procedures were performed by six surgeons via sternotomy. Cardiopulmonary bypass (CPB) was conducted with a roller pump, membrane oxygenator (Medtronic, Minneapolis, MN, USA), microembolus filter and tubing system. Packed red blood cells (PRBCs) were added to the circuit as needed to maintain hemoglobin levels above 70 g/L. Mild hypothermia cardiopulmonary bypass was performedusing non-pulsatile flow. Cold blood was used to induceand maintain cardioplegic arrest.During surgery, hemodynamic and fluid management were performed based on CVP, blood pressure and clinical evaluation of the attending anesthesiologist. After termination of CPB, residual pump blood was collected in a bag containing sodium citrate, neutralized by protamine, and returned to the patient.

**Supplementary Information 4**

**Assessment of PLA**

Arterial blood samples for assessment of PLA and TNF-α were collected after anesthesia induction. PLA was measured using flow cytometry. Briefly, blood samples were collected into an EDTA-pretreated vacutainer (BD, Basel, Switzerland) and processed within 2 hours. Red blood cells were lysed using ammonium chloride, and the remaining cells were collected and re-suspended in phosphate-buffered saline. Nonspecific binding was blocked using FcR-blocking reagents (MiltenyiBiotec, BergischGladbach, Germany). Samples were incubated in the dark for 20 minutes at room temperature with 4.0μL CD62L-PE (BD), 4.0μL CD41a-FITC (BD) and 40.0μL DRAQ5 (Biotium, Hayward, CA, USA). An IgG1-FITC/PE antibody (BD) served as an isotype control. Data were acquired using a Calibur flow cytometer (BD) equipped with FACS Diva 5.0 software and analyzed by Flowjo software (Tree Star, Ashland, OR, USA). The instrument was calibrated daily using Rainbow Beads (BD). A minimum of 200,000 events were acquired.

The boundary between positive and negative cells was determined using fluorescence-minus-one controls and an internal control. Leukocytes were gated in forward scatter/side scatter dot plots. PLA was identified using CD62L/CD41a dot plots after confirming cell nuclei using DRAQ5. Putative PLA was verified by sorting stained samples using FACSAriaand by observing samples under a confocal fluorescence microscope.

**Supplementary Information 5**

**Supplementary statistical analysis**

Distribution of continuous variables was assessed using the Shapiro-Wilk test. Normally distributed continuous data were reported as mean and standard deviation, and analyzed by unpaired or revised Student’s *t* test based on the result of Levene's test (homogeneity assessment). Skewed continuous data were reported as median (interquartile range, IQR) and analyzed using the rank sum test. Categorical data were expressed as count (percentage), and analyzed by χ^2^ or Fisher’s exact test. The comparison of baseline characteristics was used to select potential risk and confounding factors.

Cox proportional hazards regression was used to assess the association between 3-year outcomes and pre- and intra-operative variables, which included demographics (age, gender, BMI and smoking history), medical history (NYHA class, diabetes, atrial fibrillation, hypertension and left atrial thrombus), medication (warfarin, aspirin, calcium antagonists, β-blocker, digoxin, insulin, angiotensin-converting enzyme inhibitor and diuretics), preoperative blood cell counts (leukocytes, platelet and erythrocytes) and intraoperative variables(type of valve replacement, CPB time, cross-clamp time and blood transfusion). Univariate Cox proportional hazards regression analyses were performed for each variable to assess the independent risk factor, and all variables significant at the p< 0.05 level were entered into multivariate backward Cox proportional hazards regression respectively with adjusted factors. Hazard ratios (HR) for MACCE were adjusted by age, gender, body mass index, smoking history, NYHA class, diabetes, stroke history, atrial fibrillation, hypertension, digoxin use, type of valve replacement, concomitant tricuspid repair and valve material. Logistic regression was used for perioperative outcomes, and odds ratios (ORs) for perioperative AEs were adjusted by the factors described above, as well as by CPB time. The adjusted variables were chosen because they have been identified as confounders in analyses of cardio-cerebrovascular events [1]: age, sex, vascular risk factors (smoking status; the presence of hypertension, diabetes, or hyperlipidemia), other relevant variables (left ventricular ejection fraction, percutanous coronary intervention, acute coronary syndrome; the severity of coronary artery disease; angiotensin-converting–enzyme (ACE) inhibitors, beta-blockers, statins, and platelet inhibitors), vascular risk factors (body mass index, smoking history, diabetes, stroke history, atrial fibrillation, hypertension), NYHA functional class (reflecting cardiac function), digoxin, type of valve replacement and concomitant tricuspid repair (reflecting disease) and CPB time (reflecting postoperative inflammatory state).

We performed receiver operating characteristic (ROC) curves, based on MACCE and PLA, from the logistic regression model, and then Youden’s index were used to identify the optimal PLA cut-off value for predicting MACCE in 3 years. This threshold was used to classify patients as having low or high PLA, and MACCE-free survival was compared between the two groups using Kaplan-Meier analysis and the log rank test.

**Supplementary Information 6**

**Supplementary Figure**


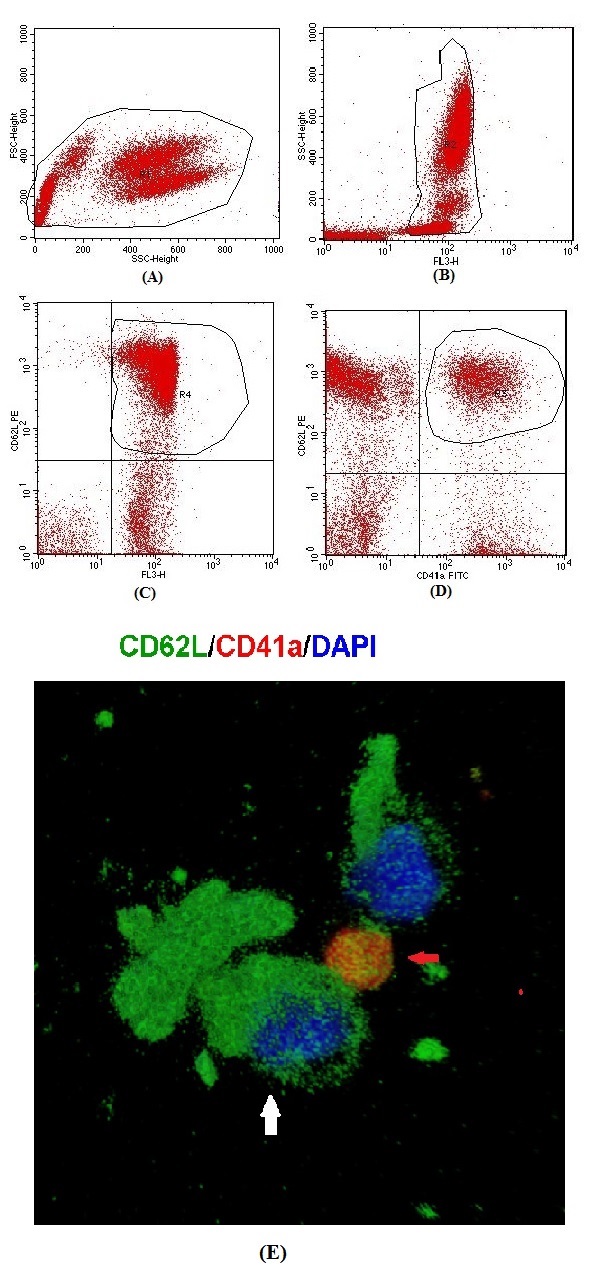


**Supplementary Figure 1. Identification of PLA by (A-D) flow cytometry and(E)confocal microscopy.**(A) Leukocytes were gated in forward scatter/side scatter dot plots. (B, C) Leukocyteswere identified using FSC/SSC/FL3/CD62L. (D) PLA was identified using CD62L/CD41a dot plots. Putative PLA was verified by sorting stained samples using FACSAria. (E) PLA was identified using CD62L/CD41a dot plots after cell nuclei were confirmed using DRAQ5 staining. Stained samples were sorted and examined using laser confocal microscopy.The red arrow points to platelets, while the white arrow points to a leukocyte. PLA, platelet-leukocyte aggregates.


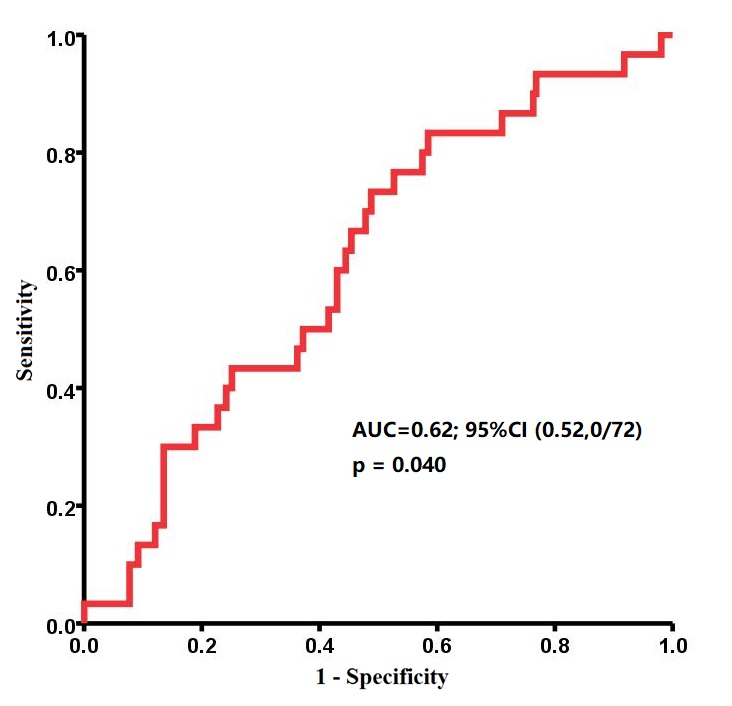


**Supplementary Figure 2. Receiver operating characteristic curve for PLA.**

**Supplementary Information 7**

**Supplementary Tables**

Supplementary Table 1 Perioperative features of patients^#^

| **Feature** | MACCE^#^ | | *p* value |
| --- | --- | --- | --- |
|  | Yes  (n=30) | No  (n=207) |  |
| **Demographics** |  |  |  |
| Age, yr | 50±8 | 47±9 | 0.118 |
| Male, n (%) | 7 (23.3) | 68 (32.9) | 0.295 |
| Body mass index, kg/m^2^ | 22.3±2.9 | 22.3±2.8 | 0.969 |
| Smoking, n (%) | 3 (10.0) | 47 (22.7) | 0.111 |
| **Medical history, n (%)** | | | |
| New York Heart Association functional class | |  | 0.603 |
| II | 3 (10.0) | 31 (15.0) |  |
| III | 27 (90.0) | 175 (85.0) |  |
| Diabetes | 1 (3.3) | 4 (1.9) | 0.495 |
| Atrial fibrillation | 15 (50.0) | 99 (47.8) | 0.824 |
| Hypertension | 3 (10.0) | 15 (7.2) | 0.870 |
| Left atrial thrombus | 4 (13.3) | 28 (13.5) | 1.000 |
| Type of valvular disease, n (%) |  |  |  |
| Mitral valve |  |  | 0.779 |
| Stenosis | 10 (33.3) | 59 (28.5) |  |
| Regurgitation | 3 (10.0) | 30 (14.5) |  |
| Stenosis and regurgitation | 14 (46.7) | 87 (43.1) |  |
| Aortic valve |  |  | 0.355 |
| Stenosis | 0 (0) | 9 (4.3) |  |
| Regurgitation | 9 (30.0) | 74 (35.7) |  |
| Stenosis and regurgitation | 13 (43.3) | 61 (30.2) |  |
| Tricuspid regurgitation | 17 (56.7) | 97 (46.9) | 0.335 |
| Echocardiographic data |  |  |  |
| [Left ventricle](javascript:;) ejection fraction, % | 61±14 | 60±13 | 0.772 |
| [Left ventricle](javascript:;) diameter, mm | 48±12 | 51±12 | 0.149 |
| Left atrial diameter, mm | 50±16 | 51±17 | 0.712 |
| Right ventricle diameter, mm | 21±6 | 21±5 | 0.738 |
| EuroSCORE, n (%) |  |  | 0.132 |
| Low (0–2) | 27 (90.0) | 194 (93.7) |  |
| Medium (3–5) | 3 (10.0) | 12 (5.8) |  |
| High (≥6) | 0 (0.0) | 1 (0.5) |  |
| **Medications, n (%)** |  |  |  |
| Warfarin | 0 (0.0) | 3 (1.4) | 1.000 |
| Aspirin | 1 (3.3) | 10 (4.8) | 1.000 |
| Calcium antagonists | 0 (0.0) | 2 (1.0) | 1.000 |
| β-blocker | 2 (6.2) | 17 (8.2) | 1.000 |
| Digoxin | 1 (3.3) | 24 (11.6) | 0.290 |
| Insulin | 0 (0.0) | 1 (0.5) | 1.000 |
| Angiotensin-converting enzyme inhibitor | 0 (0.0) | 8 (3.9) | 0.579 |
| Diuretics | 3 (10.0) | 22 (10.6) | 1.000 |
| **Blood cell count before surgery** |  |  |  |
| Leukocytes (×10^9^/L) | 4.32±1.63 | 4.75±1.62 | 0.179 |
| Platelets (×10^9^/L) | 141±47 | 137±48 | 0.690 |
| Red blood cells (×10^12^/L) | 4.16±0.47 | 4.25±0.57 | 0.404 |
| **In operating room** | | | |
| Valve replaced, n (%) |  |  | 0.892 |
| Aortic | 6 (20.0) | 35 (17.1) |  |
| Mitral | 11 (36.7) | 83 (40.5) |  |
| Aortic and mitral | 13 (43.3) | 86 (42.4) |  |
| Bioprosthetic valves, n (%) | 1 (3.3) | 14 (6.8) | 0.749 |
| Concomitant tricuspid repair, n (%) | 14 (46.7) | 94 (45.4) | 0.897 |
| Maze, n (%) | 8 (26.7) | 56 (27.1) | 0.974 |
| CPB time, min | 121±34 | 117±36 | 0.627 |
| Cross-clamp time, min | 81±31 | 79±30 | 0.653 |
| Packed red blood cell consumption, units | 0 (0,2.50) | 0 (0,1.50) | 0.426 |
| Intensive care unit stay, hours | 65 (44, 85) | 46 (41, 67) | 0.009 |
| Hospital stay, days | 12±5 | 9±3 | 0.020 |

CPB, cardiopulmonary bypass

* Seven subjects were lost to follow-up and so were excluded from data analysis.

^#^ Deaths from traffic accidents (n=2), cancer (n=2) and pulmonary infection (n=1) were excluded.

**Supplementary Table 2. Univariate and multivariate regression to assess whether TNF-α levels were associated with outcomes**

|  | Unadjusted | | Adjusted | |
| --- | --- | --- | --- | --- |
|  | HR^†^ or OR^#^ (95%CI) | *p* value | HR^†^ or OR^#^(95%CI) | *p* value |
| MACCE | 1.01 (0.74,1.39) | 0.940 | 1.01 (0.72,1.41) | 0.970 |
| Perioperative AEs | 0.93 (0.70,1.27) | 0.634 | 0.94(0.67,1.32) | 0.740 |

This analysis was based on TNF-α quartiles. AE, adverse events; MACCE, major adverse cardiac and cerebrovascular events.

^†^ HR was adjusted by age, gender, body mass index, smoking history, NYHA functional class, diabetes, stroke history, atrial fibrillation, hypertension, digoxin, type of valve replacement, concomitant tricuspid repair and valve material.

^#^OR was calculated after adjusting for all the above variables and CPB time.

**Supplementary Table 3. Univariate and multivariate logistic regression to assess risk factors for perioperative AEs**

|  | Unadjusted | | | Adjusted | |
| --- | --- | --- | --- | --- | --- |
|  | OR (95%CI) | *p* value | | OR (95%CI) | *p* value |
| Age | 1.02 (0.98, 1.06) | 0.270 | | 1.02 (0.97, 1.06) | 0.509 |
| Gender | 0.59 (0.29, 1.20) | 0.148 | | 1.12 (0.28, 4.45) | 0.871 |
| BMI | 1.09 (0.96, 1.22) | 0.175 | | 1.08 (0.95, 1.23) | 0.243 |
| Smoking history | 2.23 (1.05, 4.74) | 0.038 | | 2.06 (0.93, 4.55) | 0.074 |
| NYHA functional class | 0.95 (0.37, 2.45) | 0.907 | | 0.72 (0.26, 2.06) | 0.550 |
| Diabetes | 3.76 (0.61, 23.29) | 0.155 | | 5.87 (0.77, 44.89) | 0.088 |
| Stroke history | 1.85 (0.36, 9.54) | 0.461 | | 2.50 (0.42, 14.87) | 0.314 |
| Atrial fibrillation | 1.08(0.54, 2.16) | 0.826 | | 1.23 (0.48, 3.13) | 0.670 |
| Hypertension | 3.03 (1.06, 8.65) | 0.038 | | 1.78 (0.55, 5.74) | 0.338 |
| Digoxin | 0.42 (0.10, 1.86) | 0.254 | | 0.49 (0.11, 2.30) | 0.367 |
| Valve surgery |  | 0.079 | |  | 0.035 |
| Aortic vs. Aortic and mitral | 1.99 (0.83, 4.77) | 0.121 | | 3.30 (1.09, 10.03) | 0.035 |
| Mitral vs. Aortic and mitral | 0.69 (0.30, 1.57) | 0.372 | | 0.72 (0.31, 1.67) | 0.449 |
| Concomitant tricuspid repair | 1.38 (0.69, 2.76) | 0.366 | | 2.73 (1.09, 6.85) | 0.032 |
| CPB time | 1.00 (1.00, 1.01) | 0.352 | | 1.01 (0.99, 1.02) | 0.369 |
| valve material | 0.79 (0.21, 2.90) | | 0.718 | 1.56 (0.35, 6.85) | 0.559 |

**Supplementary Table 4. Univariate and multivariate Cox regression to identify risk factors of MACCE*.**

|  | Unadjusted | | Adjusted | |
| --- | --- | --- | --- | --- |
|  | HR (95%CI) | *p* value | HR (95%CI) | *p* value |
| Age | 1.55 (0.66, 3.61) | 0.311 | 1.03 (0.99, 1.07) | 0.159 |
| Gender | 1.03 (0.99, 1.07) | 0.129 | 0.70 (0.24, 2.07) | 0.518 |
| BMI | 1.00 (0.88, 1.13) | 0.957 | 0.99 (0.87, 1.13) | 0.884 |
| Smoking history | 0.39 (0.12, 1.28) | 0.120 | 0.39 (0.12, 1.30) | 0.126 |
| NYHA functional class | 1.51 (0.46, 4.99) | 0.496 | 1.71 (0.51, 5.71) | 0.382 |
| Diabetes | 1.53 (0.21, 11.26) | 0.674 | 1.33 (0.16, 10.89) | 0.788 |
| Stroke history | 2.66 (0.63,11.19) | 0.181 | 3.15 (0.73, 13.50) | 0.123 |
| Atrial fibrillation | 1.12 (0.55, 2.28) | 0.765 | 1.08 (0.50, 2.31) | 0.850 |
| Hypertension | 1.35 (0.41, 4.46) | 0.619 | 1.55 (0.44, 5.50) | 0.495 |
| Digoxin | 0.28 (0.04, 2.02) | 0.205 | 0.28 (0.04, 2.02) | 0.205 |
| Concomitant tricuspid repair | 1.09 (0.53, 2.22) | 0.823 | 1.06 (0.45, 2.54) | 0.890 |
| Valve surgery |  | 0.919 |  | 0766 |
| Aortic and mitral vs. Aortic | 1.11 (0.42, 2.93) | 0.827 | 0.68 (0.23, 2.02) | 0.487 |
| Mitral vs. Aortic | 0.91 (0.41, 2.03) | 0.812 | 0.68 (0.22, 2.13) | 0.510 |
| valve material | 1.93 (0.26, 14.17) | 0.518 | 2.67 (0.35, 20.71) | 0.347 |

**SupplementaryInformation 8**

**Supplementary References: references of definitions**

1.Mhutcheson. the Society for Thoracic Surgeons and the American College of Cardiology Foundation. 2015.

2.Dworschak M, Czerny M, Grimm M, Grubhofer G, Plöchl W. The impact of asymptomatic carotid artery disease on the intraoperative course of coronary artery bypass surgery. Perfusion. 2003;18(1):15-18.

3.Endorsed by the European Society of Clinical Microbiology and Infectious Diseases (ESCMID) and by the International Society of Chemotherapy (ISC) for Infection and Cancer, Authors/Task Force Members, Habib G, et al. Guidelines on the prevention, diagnosis, and treatment of infective endocarditis (new version 2009): The Task Force on the Prevention, Diagnosis, and Treatment of Infective Endocarditis of the European Society of Cardiology (ESC). Eur Heart J. 2009;30(19):2369-2413.

4.Chung KF, Wenzel SE, Brozek JL, et al. International ERS/ATS guidelines on definition, evaluation and treatment of severe asthma. Eur Respir J. 2014;43(2):343-373.

5.Postma DS, Bush A, van den Berge M. Risk factors and early origins of chronic obstructive pulmonary disease. Lancet Lond Engl. 2015;385(9971):899-909.

6.Canet J, Gallart L, Gomar C, et al. Prediction of Postoperative Pulmonary Complications in a Population-based Surgical Cohort: Anesthesiology. 2010;113(6):1338-1350.

7.Larrey D. Epidemiology and individual susceptibility to adverse drug reactions affecting the liver. Semin Liver Dis. 2002;22(02):145-155.

8.KDIGO 2012 clinical practice guideline for the evaluation and management of chronic kidney disease. | National Guideline Clearinghouse. https://www.guideline.gov/summaries/summary/46510? Accessed July 2, 2018.

9.Hunt SA, Baker DW, Chin MH, et al. ACC/AHA Guidelines for the Evaluation and Management of Chronic Heart Failure in the Adult: Executive Summary A Report of the American College of Cardiology/American Heart Association Task Force on Practice Guidelines (Committee to Revise the 1995 Guidelines for the Evaluation and Management of Heart Failure): Developed in Collaboration With the International Society for Heart and Lung Transplantation; Endorsed by the Heart Failure Society of America. Circulation. 2001;104(24):2996-3007.

10.Clements IP, Wiseman GA, Hodge DO, Jacobson AF. Outcome prediction in heart failure with atrial fibrillation: relative role of left ventricular ejection fraction and neurohormonal measures. J NuclCardiol Off Publ Am Soc NuclCardiol. 2013;20(5):821-829.

11.Okusa MD, Davenport A. Reading between the (guide)lines--the KDIGO practice guideline on acute kidney injury in the individual patient. Kidney Int. 2014;85(1):39-48.

12.García-Cabrera E, Fernández-Hidalgo N, Almirante B, et al. Neurological complications of infective endocarditis: risk factors, outcome, and impact of cardiac surgery: a multicenter observational study. Circulation. 2013;127(23):2272-2284.

13.Roussos C, Koutsoukou A. Respiratory failure. Eur Respir J Suppl. 2003;47:3s-14s.
